# Long-term changes in nitrogen and phosphorus emission into the Vistula and Oder catchments (Poland)—modeling (MONERIS) studies

**DOI:** 10.1007/s11356-018-2945-7

**Published:** 2018-08-25

**Authors:** Marianna Pastuszak, Tomasz Kowalkowski, Jerzy Kopiński, Andrzej Doroszewski, Beata Jurga, Bogusław Buszewski

**Affiliations:** 1 National Marine Fisheries Research Institute, ul. Kołłątaja 1, 81-332 Gdynia, Poland; 2 Chair of Environmental Chemistry and Bioanalytics, Faculty of Chemistry, Nicolaus Copernicus University, ul. Gagarina 7, Toruń, Poland; 3 Institute of Soil Science and Plant Cultivation, State Research Institute, ul. Czartoryskich 8, 24-100 Puławy, Poland

**Keywords:** Vistula, Oder, Nitrogen, Phosphorus, Emission, Retention

## Abstract

Modeling (MONERIS) studies allowed calculation of nitrogen (N) and phosphorus (P) emission into the Vistula and Oder basins (Poland), and facilitated estimation of N and P retention in these catchments in 1995–2015. In the discussion of results, data of other authors were used in order to get an insight into N (1880–2015) and P emission (1955–2015) into the Oder basin. Population growth and agricultural intensification were responsible for respective 5.3-fold and 3.5-fold increase in N and P emission into the Oder basin, with the maximum (135,000 tons N year^−1^; 14,000 tons P year^−1^) observed at the turn of the 1980s/1990s. Pro-ecological activities during the economic transition period (since 1989) covered various sectors of the economy including agriculture, environmental protection related to, e.g., construction of a large number of waste water treatment plants (WWTPs). Consequently, in 1985–2015, the emission into the Oder basin decreased from the abovementioned maxima to 94,000 tons N year^−1^ and to 5000 tons P year^−1^, whereas in 1995–2015, the emission into the Vistula basin decreased from 170,000 to 140,000 tons N year^−1^ and from 14,200 to 10,600 tons P year^−1^. In 1995–2015, groundwater, tile drainage, and WWTPs played a key role in N emission, while erosion, overland flow, WWTPs, and urban areas played a predominant role in P emission. The relative shares of nutrient emission pathways in overall N and P emission were considerably changing over time. Extreme weather conditions have a great impact on increased (floods) or decreased (droughts) nutrient emission; particularly, N emission is susceptible to variable weather conditions. In total, approximately 91,000 tons of N and 7600 tons of P were retained annually in the river basins.

**Electronic supplementary material:**

The online version of this article (10.1007/s11356-018-2945-7) contains supplementary material, which is available to authorized users.

## Introduction

Over the last decades, geochemistry in rivers and as well as loads of nutrients carried with rivers and supplying the coastal marine zones have undergone substantial changes on a global scale (Meyer and Turner 1992; Vitousek et al. 1997; Meybeck 2004). The twentieth century was characterized by growing population and increases in energy and food production (Bongaarts 2009). The gains in food production came at a cost, leaving a significant environmental footprint on the ecosystem (Khan and Hanjra 2008). Agriculture is perceived as the largest source of nitrogen supplied to many coastal ecosystems on the globe (Torrent et al. 2007; Howarth 2008). On both, a global and local scale, the increase in nitrogen (N) and phosphorus (P) loads in various reservoirs is associated with an increase in the use of mineral fertilizers and the intensity of animal production (Ruttenberg 2003; Erisman et al. 2013; Sharpley et al. 2013). In Europe, the supply of reactive nitrogen into the environment has more than tripled since 1900, impacting on water quality, air quality, the greenhouse gas balance, ecosystems and biodiversity, and soil quality (Sutton et al. 2011). Recent studies show that less than half of N and 30% of P introduced into the natural environment in the form of mineral and natural fertilizers is effectively used, while the rest is dispersed in the natural environment and thus contributes to various negative ecological and health effects (Galloway and Cowling 2002; Galloway et al. 2003, 2008; Howarth 2008; Erisman et al. 2013).

Hydrological water flows provide the medium for transport and biogeochemical processing, via surface runoff or flow through aquifers, streams, lakes, reservoirs, and wetlands (Bouwman et al. 2013; Harrison et al. 2009). Global water cycles, carbon energy cycle, and food production are inextricable from climate change (D’Almeida et al. 2007). Climate changes not only affect the hydrological cycle, thus modify the transformations and transport characteristics of nutrients (Bouraoui et al. 2002), but in marine environment contribute to qualitative and quantitative ecosystem changes (Möllmann et al. 2009). Long-term studies carried in Europe (Kundzewicz et al. 2013) clearly indicate an increasing trend in the number of reported floods exceeding severity and magnitude thresholds. In order to reduce flood risks, or secure navigability, many rivers have been canalized which increases nutrient and organic matter loading (Kristensen and Hansen 1994; Shore et al. 2014).

River outflow is the main source of N and P supplying the Baltic Sea. It is estimated that about 75% of the total nitrogen (TN) load and 95–99% of the total phosphorus (TP) load reach the Baltic in the river’s outflow; the rest comes from the atmosphere. Poland, with a significant riverine water outflow, and with a 45% share of agricultural land and a 50% share of population in the Baltic catchment, is responsible for significant N and P loads discharged to the Baltic Sea (Pastuszak 2012). Protection of European waters against degradation has become high on the agenda of the European Commission, and locally, e.g., the Baltic Sea, on the agenda of the Helsinki Commission (HELCOM) (HELCOM 2013; Jadczyszyn and Rutkowska 2012; EEA 2015).

Models are useful assessment tools for quantification of pollution pressures by nutrients (De Wit 2000). They are essential to improve our understanding of the interactions between multiple processes in different landscape elements in river basins and to better predict the transfer of nutrients from land to sea (Bouwman et al. 2013). Over the last decades, many different models of nutrient transport, retention, and loss in river basins have been developed and applied to European rivers (Kronvang et al. 1995; Arheimer and Brandt 1998; Dumont et al. 2005; Harrison et al. 2005; Behrendt et al. 2008; Gadegast et al. 2011; Beusen et al. 2016).

In these studies, we have applied the GIS-oriented model MONERIS, and the aims of the study are as follows: (i) updating (2009–2015) of N and P emission into the Vistula and Oder catchments and combining our data (1995–2015) with the historical data (obtained with the same model) of other researchers (Oder, 1880–2000) in order to present changes in N and P emission over the last 135 and 60 years, respectively; (ii) reference of changes in the N and P emission pathways to changes in anthropogenic pressure; (iii) estimation of N and P retention in the Vistula and Oder catchment.

## Material and methods

### Study area

With surface area of 31.268 million ha (312,683 km^2^), Poland ranks among Europe’s larger countries. In 2016, agricultural land (AL) occupied 60%, forest 30.4%, built-up areas 5.4%, land under water 2.1%, and the remaining items 2.1% of the country’s area (GUS 2017). Most of Polish farms are mixed production entities growing fodder for their own animals. In the last years, they were characterized by high share of combine harvested crops (80% of cereals and winter rape) in rotation and medium stocking of animals, on average reaching 46 livestock units (LU) per 100 ha AL (Krasowicz et al. 2012). In 2016, the rural (15.3 million) and the urban (23.1 million) population accounted for 39.3% and 60.7% of the entire population in Poland, respectively.

**Fig. 1:**
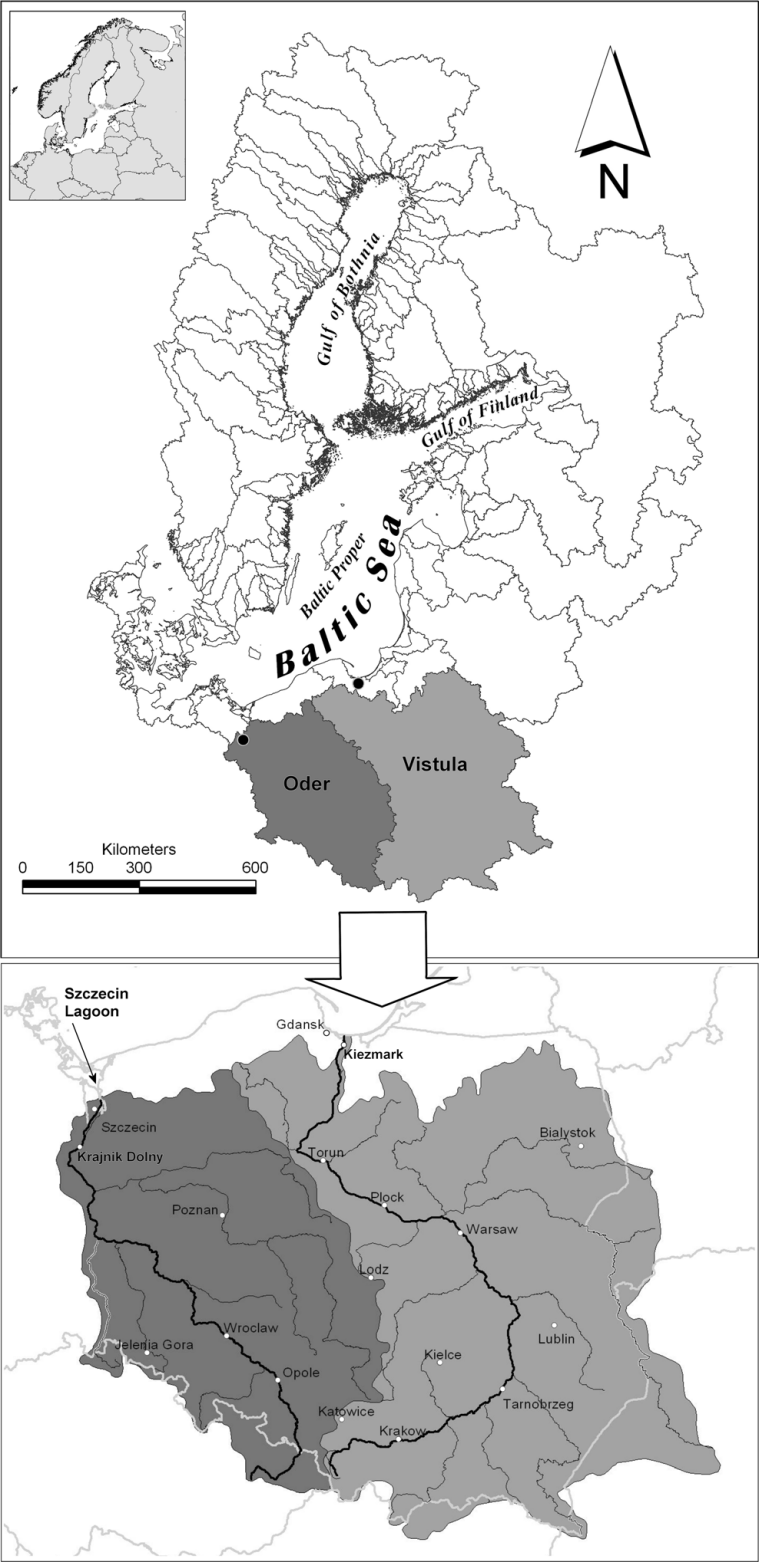
The Baltic Sea catchment and the location of investigated Vistula and Oder basins; two dots in the upper map indicate the lowermost Oder (Krajnik Dolny) and Vistula (Kiezmark) monitoring stations; the numbers indicate long-term mean annual runoff and precipitation (source: the upper part of the combined map was made available by Erik Smedberg from BNI, Stockholm University, Sweden; Fal et al. 2000; GUS 1991-2017; BDL 2017)

Almost the entire territory of Poland (99.7%) is located in the Baltic Sea drainage basin. Most of the land belongs to the large drainage basins of the Vistula River (194,424 km^2^, with 168,699 km^2^ within Polish borders, constituting ca. 54% of the territory of Poland) and the Oder River (118,861 km^2^, with 106,056 km^2^ within Polish borders, constituting ca. 34% of the territory of Poland). Small rivers drain the remaining area (> 11%) and discharge directly to the Baltic Sea (Fig. 1). The long-term (1951–1990) flow rates in the Vistula and Oder Rivers are equal to 1081 m^3^ s^−1^ and 574 m^3^ s^−1^, respectively (Fal et al. 2000). Vistula and Oder belong to the seven largest rivers in the Baltic catchment (HELCOM 2004). Polish rivers are the least regulated rivers in the Baltic region (Krasowicz et al. 2012).

### Data sources and the model calibration

The GIS (geographical information system)-oriented model MONERIS (*MO*deling *N*utrient *E*missions in *RI*ver *S*ystems) (Behrendt et al. 2000) was used to calculate nitrogen and phosphorus emission into the Vistula and Oder catchments in the period of 1995–2015. MONERIS estimates emissions of nutrients from both, point and diffuse sources. MONERIS differentiates seven pathways of nutrient emission (Behrendt et al. 2005, 2000) and uses GIS to aggregate both the input data from measurements and information calculated by the model (Pastuszak et al. 2014).

The basic input data into the model encompassed (i) mean annual N and P loads at the lowermost monitoring stations on the Vistula (Kiezmark) and the Oder River (Krajnik Dolny) in 1995–2015 (Pastuszak et al. 2018; GUS 2016), (ii) precipitation (Górski 2006) and own calculations conducted by the Institute of Soil Science and Plant Cultivation, (iii) statistical data on changes in population (BDL 2017), (iv) changes in land use (GUS 1991-2017), and (v) wastewater infrastructure with values of N and P emission from point sources (GUS 1991-2017). Values of the above parameters were calculated for the Vistula and Oder basin and GIS software (ArcView 3.3), as well as digital maps were applied in calculations.

The following data were available as geo-referenced datasets and were implemented into the model:

(i) The River Network and the catchment borders were digitized from the Atlas Podziału Hydrologicznego Polski (IMWM 2005),
(ii) The land use classification data were gathered from the CORINE Land Cover (CLC 2010), as well as from the National Statistical Office database (GUS 1991-2017),
(iii) The digital soil map was composed from the FAO map (FAO 2003),
(iv) The land elevation was obtained from the digital elevation model (DEM) with a resolution of 30 arcsec (about 925 m × 570 m, re-sampled to 500 m × 500 m) (USGS 1996),
(v) The differentiation of solid and unconsolidated rocks within the catchment areas was based on the hydrogeological map of Europe from the National Institute of Public Health and the Environment (RIVM, Holland) (own calculations),
(vi) The total nitrogen deposition in the investigated area was calculated based on the results on atmospheric deposition of nitrogen oxides and ammonium (EMEP 2017),
(vii) The borders of the administrative areas (municipalities, districts, regions, and countries) are provided with ArcView 3.3 software within “Maps and data professional sets – Europe,”
(viii) Data for calculating point source emissions between 1995 and 2015 were taken from the Main Statistical Office internet database (BDL 2017),

Nutrient balance in agriculture in the Vistula and Oder basin in 1995–2015 was calculated “on the soils surface” at the NUTS-2 level (NUTS - Nomenclature des Unités Territoriales pour des Besoins Statisques). Calculations were carried out by the Institute of Soil Science and Plant Cultivation using the OECD methodology (OECD 2004, 2006).

**Fig. 2:**
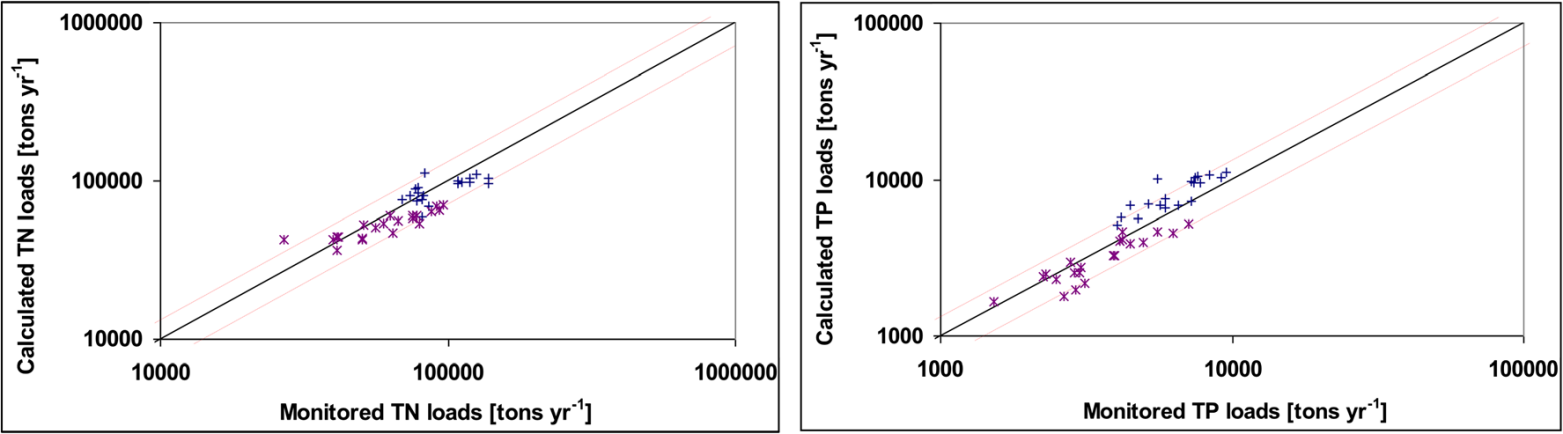
Comparison between TN and TP loads calculated with MONERIS and TN and TP mean annual loads monitored at the lowermost Vistula (Kiezmark) [blue crosses] and Oder (Krajnik Dolny) [purple asterisks] monitoring stations

Model calibration, performed for the period 1995–2015, was based on the differences between TN and TP loads calculated with MONERIS and the monitored loads at the lowermost monitoring stations on the Vistula and Oder (Pastuszak et al. 2018; GUS 2016). The absolute mean percentage error was 19% and 21% for TN and TP, respectively (Fig. 2). In addition, Nash-Sutcliffe model efficiency coefficients (Nash and Sutcliffe 1970) were calculated for TN and TP and they amounted to 0.67 and 0.63, respectively.

### Calculation of N and P retention in the Vistula and Oder basin

Nitrogen and phosphorus loads estimated at the lowermost monitoring stations can be subtracted from overall N and P modeled emission for the Vistula and Oder basin, and in that way, we obtained N and P retention in the subjected river basins. The mean and maximum and minimum retention values, expressed in percentages and tons, are presented in the “Results” section. The following formula was used in calculations:

$$ R=\frac{E_x- Lx}{E_X}\times 100\% $$

where

*R*: N and P retention [%];
*E*_*x*_: overall emission of N and P in the year *x* [tons year^−1^];
*L*_*x*_: load of TN and TP in the year *x* [tons year^−1^].

## Results

### Long-term water outflows from Polish territory to the Baltic Sea

In 1975–2015, the annual water discharge from Polish territory to the Baltic Sea varied from ca. 40 to ca. 90 km^3^. There can be distinguished four wet periods: at the turn of the 1970s and 1980s, in the late 1980s, in 1997–2002, and in 2010–2011, and four very dry periods: in 1990–1993, 2003–2009, 2012, and 2015. The difference in riverine water outflow between dry and wet periods amounted to ca. 40 km^3^. With the exception of 2012 and 2013, the Oder catchment experienced slightly lower precipitation than the Vistula catchment. Because of the larger catchment area, the Vistula drains almost twice as much water annually as the Oder. A record high water outflow, reaching ca. 25 km^3^ in the Oder and ca. 54 km^3^ in the Vistula, was observed in 2010 (Suppl. 1, 2).

### Annual (1995–2015) emission of nitrogen and phosphorus into Vistula and Oder basins

#### Nitrogen

Kowalkowski et al. (2012) divided the period of studies (1995–2008) into two sub-periods, i.e., 1995–2002 and 2003–2008. The data from sub-period 2003–2008 were used by Pastuszak et al. (2014) as a reference material in modeling of future scenarios. In order to be consistent with these elaborations, in this work, we wish to keep the same division and add the third sub-period, i.e., 2009–2015 characterized by extreme weather conditions (flood, droughts) (Table 1; Suppl. 1).

**Table 1:** Average annual emission of nitrogen and phosphorus into the Vistula and Oder basin in 1995–2002, 2003–2008, and 2009–2015; overall averages in 1995–2015

Period	Vistula basin	Vistula basin	Oder basin	Oder basin
	N [tons year^−1^]	P [tons year^−1^]	N [tons year^−1^]	P [tons year^−1^]
1995–2002	170,491	14,232	108,380	7154
2003–2008	143,270	10,651	90,188	4881
2009–2015	140,597	10,676	94,550	5051
Average	151,452	11,853	97,706	5695

**Fig. 3:**
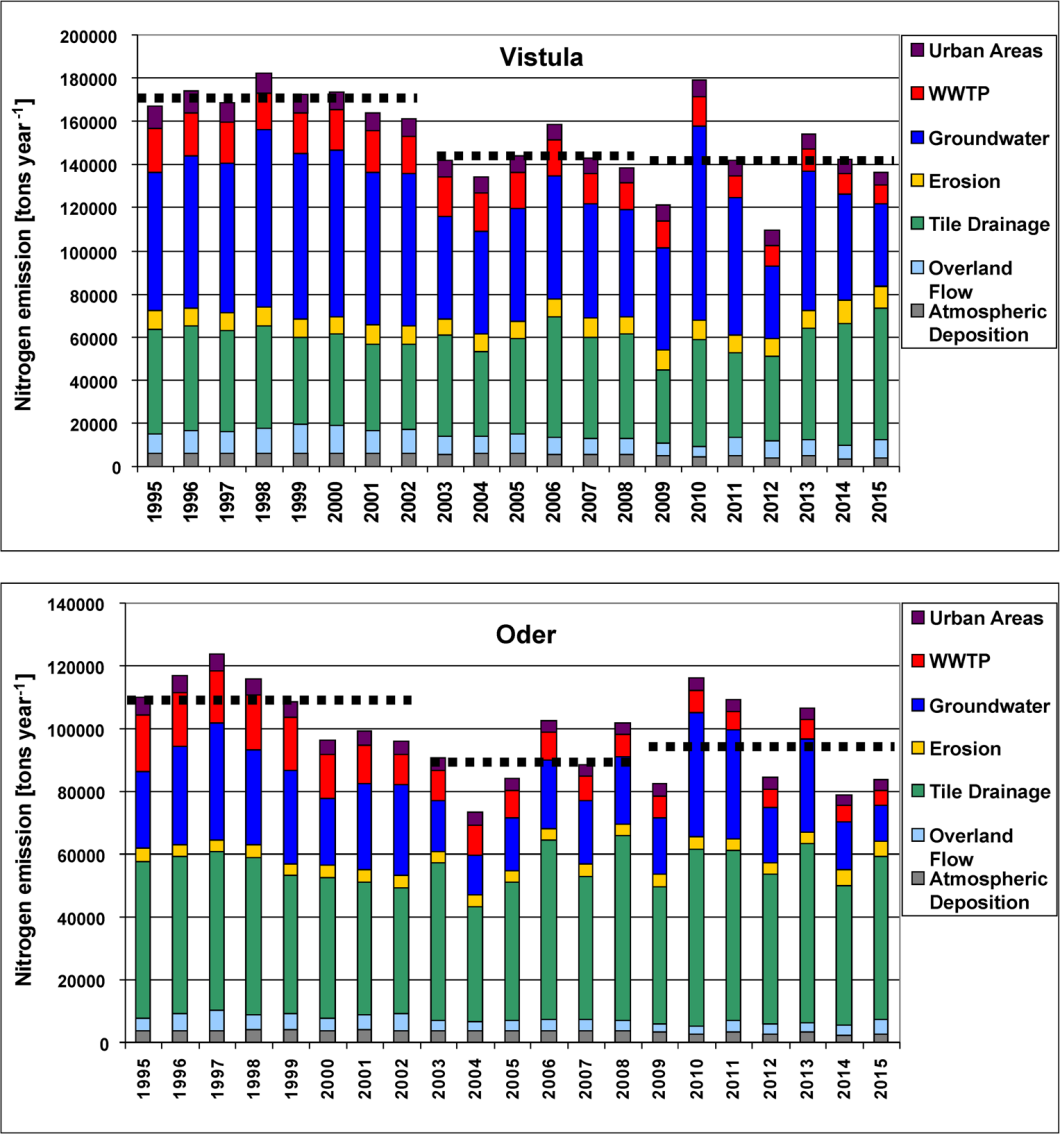
Annual source apportioned nitrogen emission into the Vistula and Oder basins in 1995–2015; horizontal broken lines show the average N emission calculated for the periods 1995–2002, 2003–2008, and 2009–2015 (please note different scales)

The average annual N emission, calculated for three sub-periods, was (i) by ca. 54,000 tons year^−1^ higher in the Vistula than in the Oder basin, (ii) between the first and the second sub-period, N emission declined by ca. 27,000 tons in the Vistula and by ca. 18,000 tons in the Oder basin (Table 1). The declines were mainly generated by groundwater pathway (29% Vistula, 37% Oder), by WWTPs (15% Vistula, 44% Oder), and by overland flow (27% Vistula, 32% Oder). The third sub-period was characterized by the fluctuations in N emission, with the highest values observed in both basins in 2010 (flood), approaching those from the 1990s, and with the average N emission slightly lower in the Vistula and slightly higher in the Oder basin as compared with values in the second sub-period (Fig. 3; Suppl. 1–4).

**Fig. 4:**
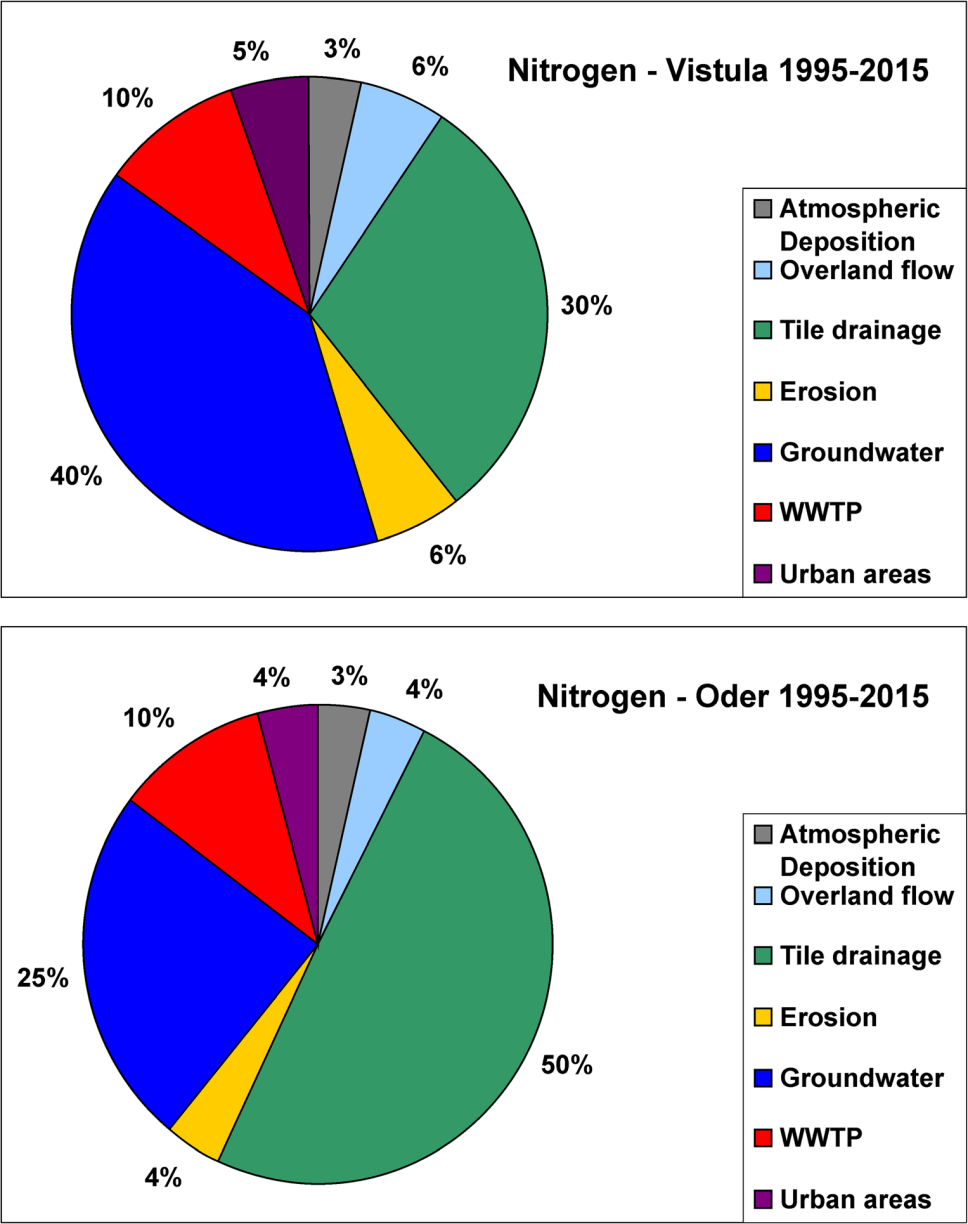
Percentage contribution of seven nitrogen pathways in the Vistula and Oder basin in 1995–2015

Three N pathways, i.e., groundwater, tile drainage, and to a lesser extent WWTPs, played a key role in N emission, and they were responsible for ca. 80% of overall N emission into both basins in 1995–2015 (Fig. 4). Contribution of groundwater pathway was particularly high in the Vistula basin in 2010 (Suppl. 3, 4). In 1995–2015, N emission via groundwater was by fifteen percentage points higher, whereas N emission via tile drainage was by twenty percentage points lower in the Vistula than in the Oder basin, and this general finding holds for the three sub-periods studied (Fig. 4; Suppl. 3, 4).

#### Phosphorus

**Fig. 5:**
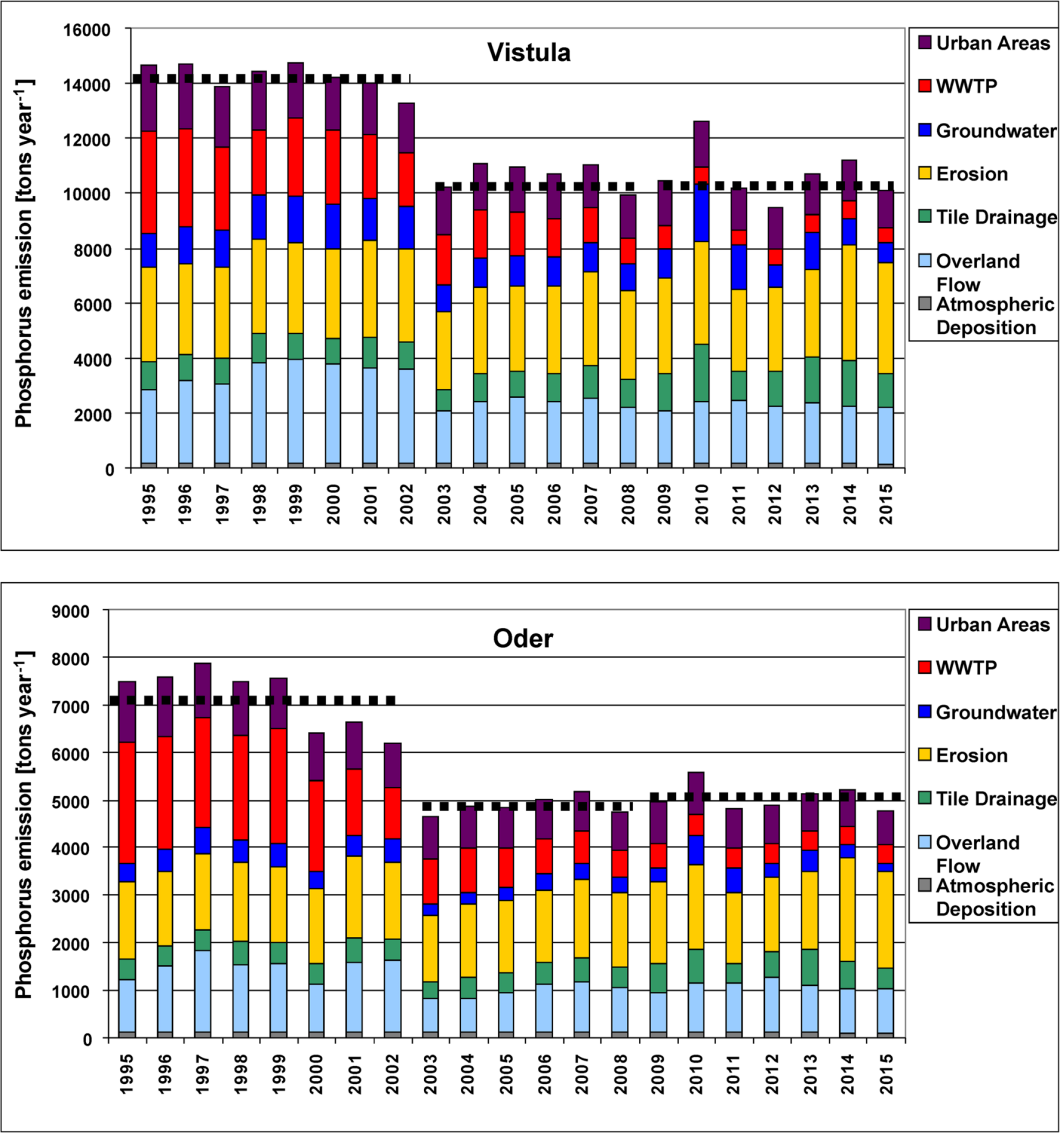
Annual source apportioned phosphorus emission into the Vistula and Oder basins in 1995–2015; horizontal broken lines show the average P emission calculated for the periods 1995–2002, 2003–2008, and 2009–2015 (please note different scales)

**Fig. 6:**
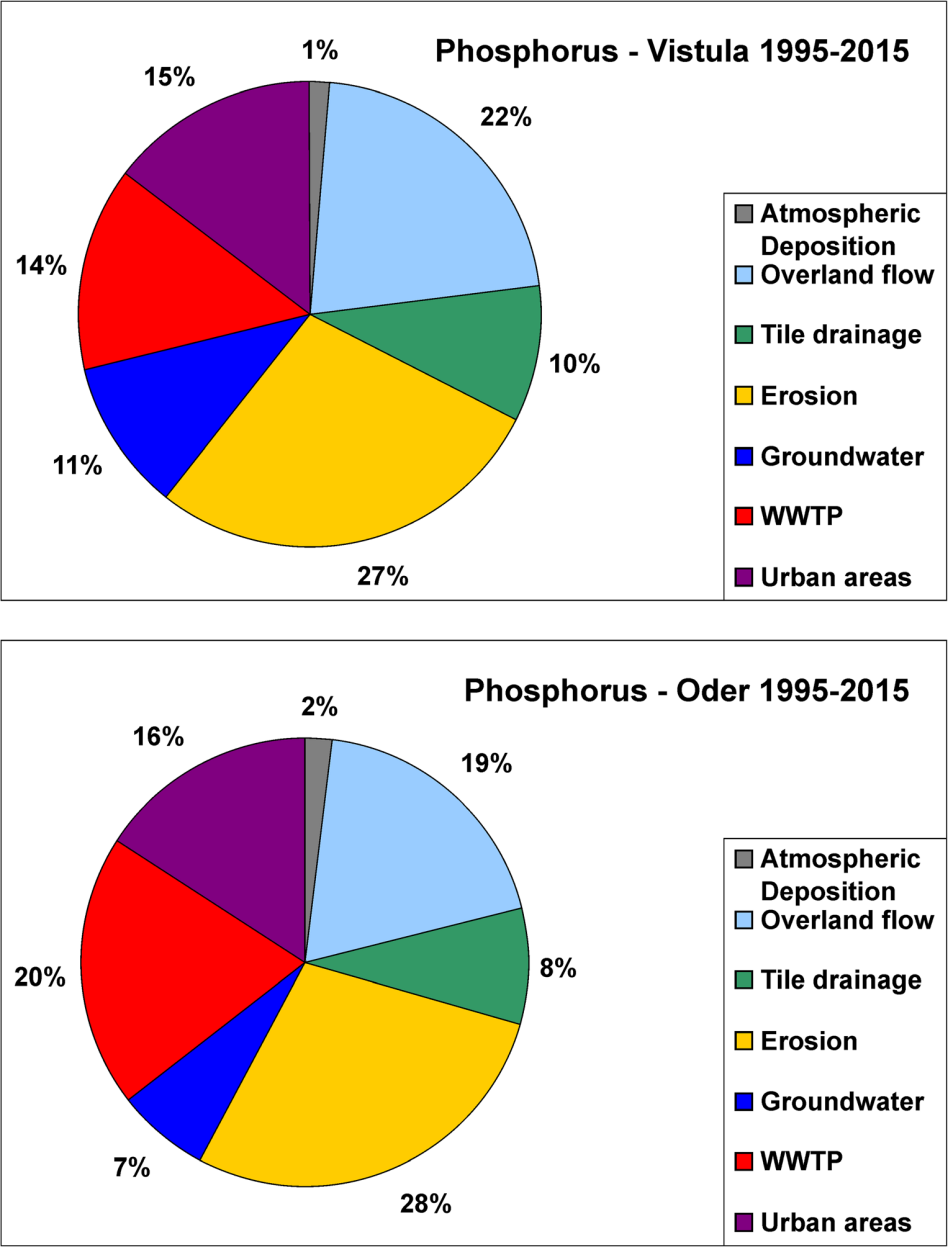
Percentage contribution of seven phosphorus pathways in the Vistula and Oder basin in 1995–2015

The average annual P emission, calculated for three sub-periods, reached 11,853 tons year^−1^ in the Vistula basin and it was ca. two times higher than in the Oder basin (Table 1). Between the first and the second sub-period, P emission declined by 25% (ca. 3600 tons) in the Vistula and by 32% (ca. 2300 tons) in the Oder basin, and the declines were mainly generated by WWTPs (48% Vistula, 61% Oder), overland flow (34% Vistula, 37% Oder), and groundwater pathway (30% Vistula, 36% Oder). The third sub-period was characterized by (i) high fluctuations in P emission, with the highest value observed in 2010 (flood) in both basins, while the lowest in 2012 (Vistula) and in 2015 (Oder) (droughts); (ii) in the third sub-period, the average P emission was either comparable (Vistula) or slightly higher (Oder) than in the second sub-period (Fig. 5; Table 1). Four pathways, i.e., erosion, overland flow, WWTPs, and urban areas, played a key role in P emission and accounted for ca. 80% of the total P emission in both basins. In 1995–2015, the average percentage contribution of WWTPs pathway was by six percentage points higher in the Oder than in the Vistula basin (Fig. 6; Suppl. 5, 6).

### N and P retention in the Vistula and Oder basin

The mean percentage of N and P retention, as well as the mean annual retention expressed in tones, was higher in the Vistula than in the Oder catchment. In 1995–2015, the mean N retention in both river systems reached 91,393 tons N year^−1^ and 7637 tons P year^−1^. There are quite substantial differences between the maximum and the minimum retention values in both river basins (Table 2).

**Table 2:** Estimated nitrogen and phosphorus retention in the Vistula and Oder catchment in 1995–2015

	Nitrogen [%]		Phosphorus [%]	
	Vistula	Oder	Vistula	Oder
Mean	37.4	34.7	46.5	35.3
Min	14.0	17.5	27.0	9.8
Max	53.5	54.5	62.1	54.1
	Nitrogen [tons N year^−1^]		Phosphorus [tons P year^−1^]	
	Vistula	Oder	Vistula	Oder
Mean	57,440	33,953	5633	2004
Min	22,572	16,791	2909	773
Max	95,681	51,027	9120	3374

## Discussion

The marine ecosystems coexist with the terrestrial ecosystems in an ecological sense and should remain in equilibrium in the sense of their goods and services. Modeling studies performed in the Baltic Sea by Håkanson et al. (2010) were based on a big long-term database and the outcome proves that adoption of improper nutrient load reduction may worsen the goods and services of the Baltic ecosystem and, moreover, can make unmanageable emission reduction requirements from the land system, as indicated by Pastuszak et al. (2018).

### Long-term variations in N and P emission into the Vistula and Oder basin

**Fig. 7:**
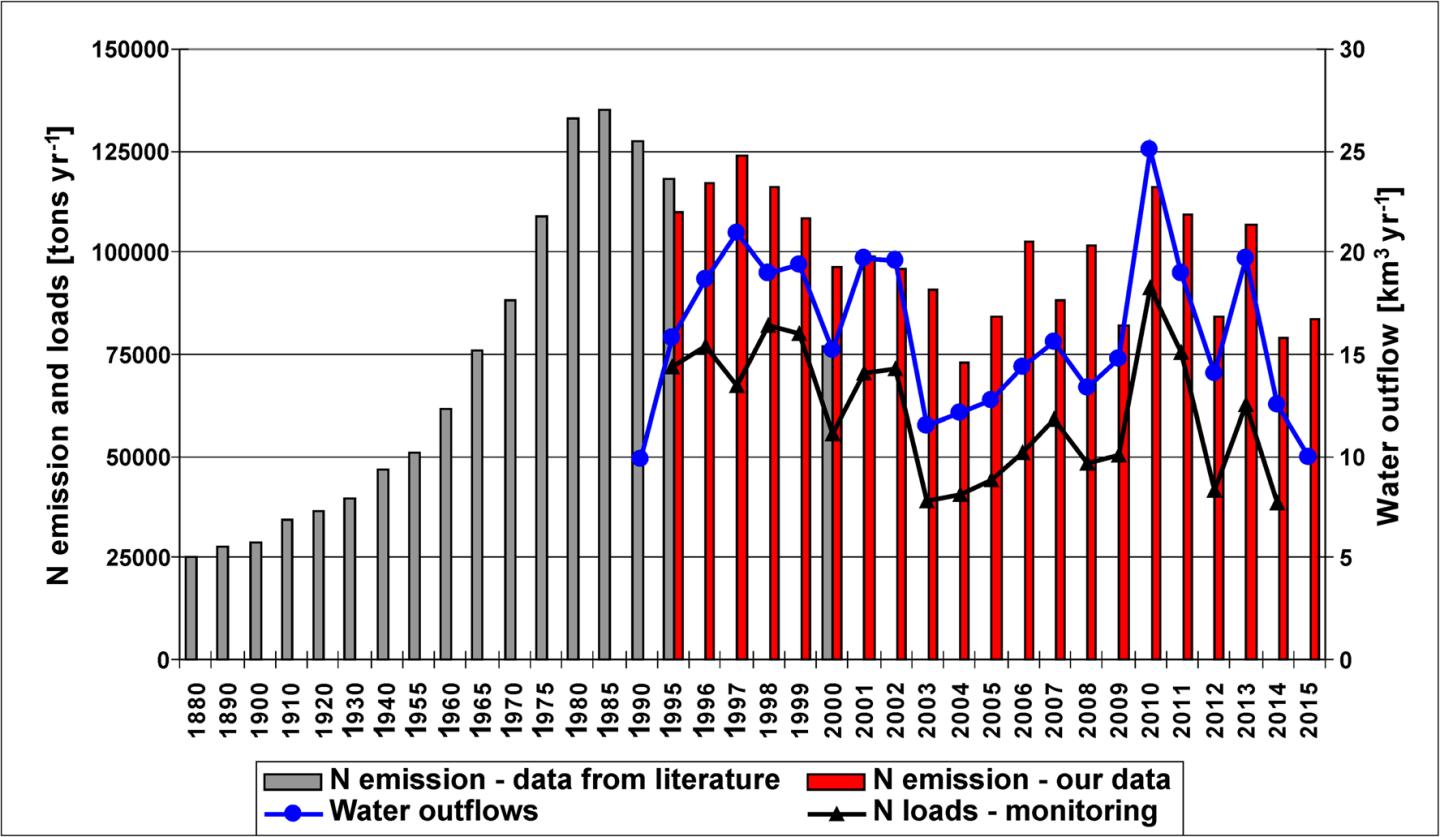
Annual N emission into the Oder basin in 1880–2015, monitored N loads discharged into the Baltic Sea in 1995–2014, water outflow by the Oder River in 1990–2015 (sources:1880–1940—Gadegast et al. 2011; 1955–2000—Behrendt et al. 2008; 1995–2015—our data; monitored nitrogen loads—Pastuszak et al. 2018; water outflows—IMWM 1989-2001, 1990-2002, 2002-2016) (please note different time intervals on the X axis)

**Fig. 8:**
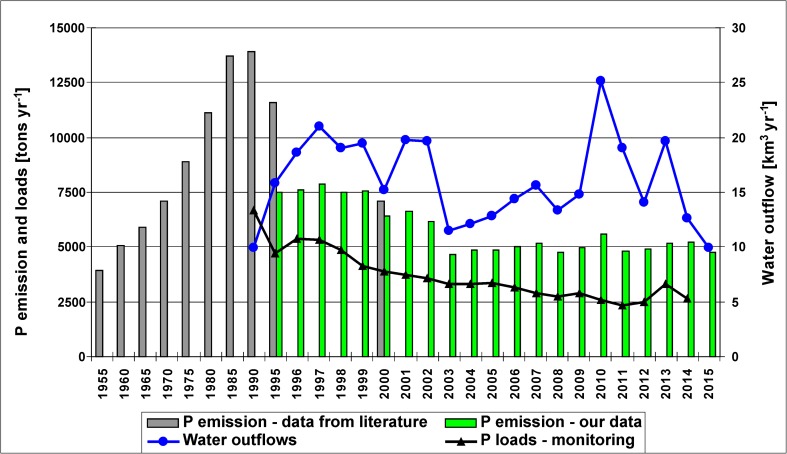
Annual P emission into the Oder basin in 1955–2015, monitored P loads discharged into the Baltic Sea in 1995–2014, water outflow by the Oder River in 1990–2015 (sources: 1955–2000—Behrendt et al. 2008; 1995–2015—our data; monitored phosphorus loads—Pastuszak et al. 2018; water outflows—IMWM 1989-2001, 1990-2002, 2002-2016) (please note different time intervals on the X axis)

The combined historical (Gadegast et al. 2011; Behrendt et al. 2008) and our data cover a period of 135 years (1880–2015) in the case of N emission, and 60 years (1955–2015) in the case of P emission into the Oder basin (Figs. 7 and 8). N and P emission into the Vistula covers the period 1995–2015 and it is referred to studies of Kowalkowski and Buszewski (2006) performed for the early 1990s.

*N emission* into the Oder basin increased from 25,000 tons year^−1^ in 1880 to 135,000 tons year^−1^ in 1985 (Gadegast et al. 2011; Behrendt et al. 2008). That maximum was followed by a declining trend in N emission which continued till 2004 in both basins, when it reached 74,000 tons year^−1^ in the Oder and 130,000 tons year^−1^ in the Vistula basin (Figs. 3 and 7). The years which followed were characterized by fluctuating N emission into both river basins, with minima in dry years and maxima in wet years. The patterns of water outflow and nutrient loads discharged to the Baltic Sea are very similar in the case of N but quite different in the case of P (Figs. 7 and 8). This points to increased mobility of nitrogen both on the soil profile and horizontally with increasing and long-lasting rainfall, e.g., in 2010/2011 (flood); high correlation coefficients for nitrogen loads vs. water runoff confirm this statement (Suppl. 1, 2; Suppl. Table S1). The share of groundwater pathway in overall N emission nearly doubled in both river basins in 2010 and 2011 (Fig. 3).

Nitrate, the most abundant form of nitrogen in riverine outflow from agriculturally active catchments, such as the Vistula and Oder basins (Pastuszak and Witek 2012), is completely water soluble and thus moves with the water until it re-enters the available soil pool, is utilized by microbes or plants, becomes denitrified, is possibly deposited or buried, or enters and possibly degrades surface and/or groundwater (Erisman et al. 2013). Studies of Meisinger and Delgado (2002) show that nitrate leaching rates are affected by rain, irrigation, tile drainage, and water table fluctuations during the growing season. Movement of nitrate with percolating water through the unsaturated zone can be very slow and time required for the present-day inputs of nitrates to reach the groundwater may be from a year to 20 years (Follett 2008; Haag and Kaupenjohann 2001; Behrendt et al. 2005). Modeling studies conducted in the Oder basin show that the groundwater fluxes changed from 350 kgN km^−2^ year^−1^ in the 1980s to 180 kgN km^−2^ year^−1^ in the 1990s (Dannowski et al. 2002).

Floods alternate with severe droughts and such events occurred in large regions of Europe, Poland included (Karsten et al. 2011). During our studies, Poland has been flooded twice: in 1997 (Mohrholz et al. 1998) and in 2010/2011 (Kundzewicz et al. 2013; Pastuszak et al. 2018). These two floods were significantly different with regard to the duration and amount of water discharged. In the recent years, droughts in Poland appear more often and it is believed that this is an effect of climate change (Doroszewski et al. 2012, 2014; Somorowska 2016). Particularly strong droughts were recorded in 2003, 2006, 2012, and 2015 (Somorowska 2016; Laaha et al. 2017; http://old.imgw.pl/klimat/). The climatic water balance (CWB) is one of the indicators used in Poland for meteorological drought monitoring and the assessment of its intensity (Łabędzki and Bąk 2014). CWB describes the interaction of energy and water (Stephenson 1990), and it is calculated as the difference between precipitation total and the reference evapotranspiration total in a particular period (Łabędzki and Bąk 2014; Doroszewski et al. 2012). Great spatial differences in CWB were identified in Poland not only in 2015, but also in other years, e.g., in 2012 (http://www.susza.iung.pulawy.pl; http://old.imgw.pl/klimat). That fact may explain great difference in N emission into the Vistula basin in 2012 and 2015 (Fig. 3), both dry but with entirely different CWB characteristics (http://www.susza.iung.pulawy.pl).

*P emission* into the Oder basin increased from 4000 tons year^−1^ in 1955 to its maximum of ca. 14,000 tons year^−1^ in 1990; that maximum was followed by a significant drop in P emission to ca. 7000 tons year^−1^ in 2000 (Behrendt et al. 2008) and to 4600 tons year^−1^ in 2003 (Figs. 5 and 8). Without a long-term perspective, we could conclude that during the transition period, covering only the years 1995–2015, P emission into the Oder basin declined by ca. 2500 tons year^−1^, while in a longer time span (1985–2015), it decreased by more than 9000 tons year^−1^ (Fig. 8), and currently, it is at the level of the emission in the 1960s. In the Oder basin, P emission leveled off in 2003–2015 (Fig. 8), but monitored phosphorus loads feeding the Baltic Sea continued to decline (Pastuszak et al. 2018), pointing to increasing retention in the Oder River system at reduced anthropogenic pressure. Indeed, P retention accounted for 30% of P emission in the 1990s and for 50% in the last 8 years (Fig. 8). Increasing ecosystem P retention under reduced anthropogenic pressure has been reported for river estuaries (Howarth et al. 1996). In the Vistula basin, the highest P emission (ca. 14,700 tons year^−1^) was recorded in the 1990s, whereas the lowest (ca. 9500 tons year^−1^) was observed in 2012 (Fig. 5). Comparison of our findings with the outcome of modeling studies, performed by Kowalkowski and Buszewski (2006) in the Vistula basin for the early 1990s, indicates that we may have observed the maximum of P emission ever recorded in this river basin.

Floods of 1997 and 2010/2011 resulted in 10–15% higher P emission as compared with values observed in the neighboring years (Fig. 5), but that impact was less pronounced than in the case of nitrogen because phosphorus is less susceptible to leaching on the soil profile (Sharpley et al. 1999, 2006). Phosphorus is particle reactive and well over 90% of the phosphorus delivered by rivers to the ocean is as particulate P (Meybeck 1982; Melack 1995). Some fraction of river suspended sediment releases its phosphorus to seawater and it is estimated that from 25 to 45% of phosphorus can be released from this source and become bioavailable in marine environment (Ruttenberg 2003).

#### Causes of long-term changes in N and P emission and in shares of N and P pathways related to river basin features

Long-term changes in N and P emission into the river basins, and the shares of N and P pathways (Figs. 3, 5, 7, and 8; Suppl. 3–6) strongly depend on anthropogenic factors, i.e., population dynamics and related loads from WWTPs, intensity of agricultural production (arable land area, tile drainage network, fertilization intensity, N and P surplus), and by natural factors (volume of water outflow, contribution of lake area, contribution of high porosity bedrock which, by its nature, favors vertical water infiltration and influences water residence time thus nutrient transformation) (Oenema and Roest 1998; Oenema et al. 2003; Lepistö et al. 2006; Kowalkowski et al. 2012). Correlation coefficients for TN and TP loads vs. water runoff are the highest, but it must be remembered that this phenomenon is typical for lowland catchments (Stålnacke et al. 1999). The other parameters which strongly influence N and P emission are arable and tile-drained land shares, WWTPs, and precipitation (Table S1).

Kiedrzyńska et al. (2014) statistically evaluated 23 catchment factors that determined TN and TP loads discharged to the Baltic Sea and concluded that these loads are positively related to the number of pigs and the human population associated with WWTPs per square kilometer; the number of cattle and agricultural area were found to influence N rather than P loads. In Poland, all these driving forces have been changing in a long time span. Since 1946, human population has increased by 14 million (Suppl. 7); population in the Oder basin increased from 9 million in the 1880s to 12 million in 1940 and 12.9 million at present (Gadegast et al. 2011; BDL 2017). The intensification of agriculture, including both the production of plants and animals (Nausch et al. 1999; Gadegast et al. 2011; Suppl. 8, 9), has become a fact. The deeper changes in this respect occurred in western Poland (Odra basin), the region that has been historically characterized by the larger holdings and more intensive agricultural activity (Fotyma et al. 2012; Igras and Fotyma 2012). The number of pigs in Poland increased from 3000 × 10^3^ in 1946 to the maximum of 22,000 × 10^3^ in 1992 and then declined to stabilize at ca. 12000 × 10^3^ in 2012–2014. The number of sites with intensive pig raising, thus higher production of manure, has been larger in the Oder basin (Kowalkowski et al. 2012). The number of cattle increased from 4000 × 10^3^ in 1946 to the maximum of ca. 14,000 × 10^3^ in the 1970s, and then continually declined to reach the level of 6000 × 10^3^ over the last decade (GUS 1981-1989; 1991-2017; Suppl. 9). The decline in livestock population was the result of legal regulations of the European Union and the meat market situation in Poland and abroad (Fotyma et al. 2012; Igras and Fotyma 2012), and caused 20% decline in manure application (Suppl. 8). A huge improvement of infrastructure in farming (e.g., construction of platforms for manure storage and covered tanks for liquid manure storage, use of spreaders for manure application) accompanied by implementation of good agricultural practices also contributed to a decline in environmental pressure generated by agricultural sector (Lipiński 2012; Pietrzak 2012).

The potential impact of applied mineral and natural fertilizers on natural environment is evaluated by the calculation of N and P surplus in agricultural sector (Fotyma et al. 2012; Igras and Fotyma 2012) (Suppl. 10). In 1960, nitrogen surplus in the Vistula and Oder basin was as low as ca. 10 kg ha^−1^ UAA year^−1^ and it increased to over 70 kg ha^−1^ UAA year^−1^ in the Oder basin in 1990s. (Suppl. 10). Though high, nitrogen surplus in Polish agriculture in 1960–2000 was up to two times lower than that in other Baltic countries under transition, and it was much lower than in countries of Western Europe, characterized by very intensive agricultural activity (GUS 1981-1989; 1991-2017; OECD 2001; Campling et al. 2005; Eriksson et al. 2007; Behrendt et al. 2008). At the same time, the nitrogen efficiency in Poland (defined as the ratio of the crop nitrogen uptake to the total input of nitrogen fertilizer), was among the highest determined for numerous European and other world countries (OECD 2001). An increase in N surplus after 2007 (Eriksson et al. 2007; Suppl. 10) is, however, of concern because there is a potential threat that N emission from this source may increase, particularly under changing climate and occasional torrential rain (Pastuszak et al. 2014). Phosphorus surplus in Poland showed maximal values of ca. 17 kg ha^−1^UAA in the 1980s and then declined to reach ca. 0.5–3 kg ha^−1^ UAA in the last years (Suppl. 10). P surplus in Polish agriculture remains at a low level, so there is no risk of increased P emission from agriculture in the coming years (Pastuszak et al. 2014).

WWTPs, the other important source of N, and particularly P emission (Figs. 3, 4, 5, and 6; Suppl. 4, 6), have been given special attention during the transition period and Poland has invested large amounts of money in construction of modern, efficient WWTPs (GUS 1981-1989; 1991-2017). At present, nearly half of the sewage volume is treated with tertiary treatment technology (Suppl. 11). A tenfold decrease in volume of untreated sewage discharged to the soil and surface waters has been a result of construction of 2580 WWTPs in 1999–2014, resulting in turn in a considerable decline in N and P loads discharged from this source (BDL 2017; Suppl. 12). In 2016, 95% of the urban population and 40% of the rural population was connected to WWTPs (GUS 1991-2017). The declines in overall nutrient emission must also be attributed to reduction of N and P emission from dispersed farmyards through construction of domestic wastewater treatment plants, serving up to 50 inhabitants. Up to the year 2010, 82,632 domestic sewage treatments were constructed in Poland, and it is anticipated that in the years that follow, 587,000 new ones were/will be constructed (Przydomowe oczyszczalnie 2012); these data are not covered by the official statistics. Considerable decline in share of WWTPs in N and P emission (Figs. 4 and 6) can be additionally explained by declines in water consumption (GUS 1981-1989; 1991-2017; Suppl. 13), and economical water utilization, which resulted in a twofold decrease in volume of industrial and municipal sewage (GUS 1981-1989; 1991-2017; Suppl. 11). Phosphorus from detergents is a significant P source in riverine outflow; P used in detergents has been practically reduced to zero in Germany and by half in Poland and Czech Republic (Behrendt et al. 2008). The withdrawal of detergents based on phosphorus compounds may be one of the ways to effectively reduce emissions of this element in Poland. Additionally, a large number of “hot spots” has been eliminated due to closure of obsolete factories and modernization of the remaining ones (HELCOM 2009).

Significantly changing anthropogenic pressure over the past hundred or more years was reflected not only in magnitude of the N and P emissions to the basins of the studied rivers, but also in the percentage share of emission paths (Suppl. 3, 5). The declines of N emission via groundwater, WWTPs, atmospheric deposition, overland flow, and urban areas have been observed in both river basins; an increase in N emission via groundwater in the third sub-period is connected with the great flood of 2010/2011 (Suppl. 2, 3). The decline in P emission via WWTPs has been the most pronounced, but the declines via overland flow and urban areas were also considerable (Suppl. 5).

**Fig. 9:**
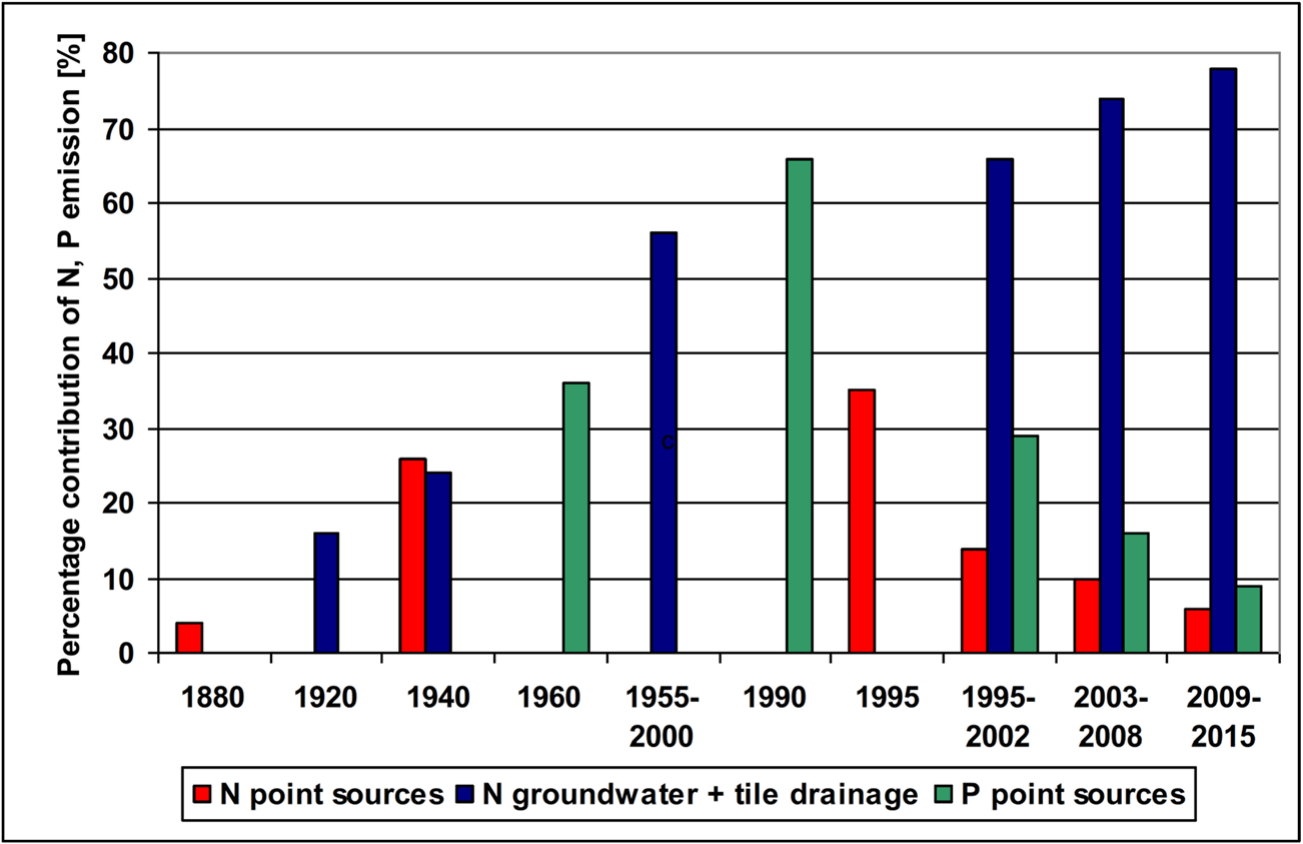
Percentage contribution of selected nitrogen and phosphorus pathway emission into the Oder basin in 1880–2015 (source: Gadegast et al. 2011; Behrendt et al. 2008; our data presented in this paper)

The long-term data (1880–2015) allow to conclude that in the Oder basin, the share of point sources in N emission reached its maximum of 35% in 1995 and then declined to 6% in 2009–2015; P emission via point sources increased from 36% in 1960 to 66% in 1990 and then declined to 9% in 2009–2015 (Fig. 9; Suppl. 4, 6). In 1965–1995, point sources were a dominant P pathway in the Oder basin (Behrendt et al. 2008). At a significant decline in percentage contribution of N and P emission from point sources, the relative contribution of agriculture increases (Fig. 9). However, flow normalized TN, and particularly TP loads, showed a continual declining tendency in both rivers in 1988–2014. In riverine outflow, P-PO_4_ mainly originates from point sources, whereas Other P (Other P = TP – P-PO_4_) from diffuse sources (Pastuszak and Witek 2012). In 1988–2014, nearly threefold decline in P-PO_4_ and in Other P concentrations in the Oder River, and a considerable decline in the Vistula River, is reported by Pastuszak et al. (2018) and that must have resulted not only from effective P removal from WWTPS (Suppl. 12), but also from implementation of legal regulations, e.g., good agricultural practices, in agricultural sector, and improvement of infrastructure in Polish agricultural holdings (Jadczyszyn and Rutkowska 2012; Król 2015). The other river monitoring data also point to a significant improvement of water quality in the Vistula and Oder (lowermost monitoring stations), with the average biological oxygen demand (BOD_5_) dropping from 5.8 to 2 mg dm^−3^ in the Vistula, and from 6.3 to 3.5 mg dm^−3^ in the Oder (Pastuszak et al. 2018).

In 1995–2015, three pathways, i.e., groundwater, tile drainage, and to a lesser extent WWTPs, played a key role in N emission, and they were responsible for ca. 80% of overall N emission in both basins. Four pathways, i.e., erosion, overland flow, WWTPs, and urban areas, played a key role in P emission and accounted for ca. 80% of overall P emission into both basins (Figs. 4 and 6). The combined data (1995–2015) show that N emission via groundwater was by fifteen percentage points higher, whereas N emission via tile drainage was by twenty percentage points lower in the Vistula than in the Oder basin, and this general finding holds for the three sub-periods studied (Fig. 4; Suppl. 4). The explanation lies in the fact that the Vistula basin is characterized by more permeable bedrock (favoring vertical water infiltration) and by less developed drainage system (to a great extent, responsible for lateral nutrient transport) as compared with the Oder basin (Kowalkowski et al. 2012).

In 1995–2015, erosion, overland flow, WWTPs, and urban areas were the main contributors to P emission into the Vistula and Oder basin (Fig. 6; Suppl. 6), with WWTP contribution dropping from 20 to 6% in the Vistula, and from 29 to 9% in the Oder basin (Suppl. 5, 6). This leads to a conclusion that there is still a small pool of N and P that can be removed from the systems by construction of WWTPs, an approach that is incomparably cheaper than mitigation measures introduced in agricultural sector (Håkanson et al. 2010).

### Area-specific export of TN and TP—comparison with other regions

Modeling studies, e.g., MONERIS, allow estimating TN and TP exports to rivers; therefore, they do not take into account the nutrient retention in the rivers themselves, whereas monitoring measurements allow determining river exports of nutrients to coastal zones. Hence, these results cannot be directly compared with each other. The results of both approaches are presented in Table 3, and the difference between the MONERIS fluxes and the monitored fluxes approximates TN and TP retention (Vistula, Oder, Elbe, Ems, Rhine, Weser) (see also to the next section.). Both modeled and monitored TN exports by the Vistula and Oder are 2–4 times lower than exports by rivers in Western Europe or on the northeast coast of the USA. TP exports by the Vistula and Oder are 3–4 times lower than those from the northeast coast of the USA or from the North Sea continental shelf, and as much as seven times lower than fluxes from the Amazon basin (EEA 2005; Radach and Pätsch 2007; Howarth et al. 1996; Table 3). A worldwide analysis performed for 946 rivers in the case of nitrogen and 685 rivers in the case of phosphorus (Alvarez-Cobelas et al. 2008, 2009) indicates that (i) TN export spans four orders, whereas TP exports span six orders of magnitude; (ii) N and P export showed a left-skewed distribution (nearly 50% of all observations represent the lowest TN and TP area-specific loads), which, as stated by the authors, suggests a relatively pristine conditions for most systems; and (iii) a case-by-case approach, based on long-term local and regional studies, appears more fruitful for predicting global N and P export; regional studies are of great importance with respect to proper nutrient management. Area-specific TN and TP fluxes by the Vistula and Oder safely fall within the most common range of the lowest fluxes reported by Alvarez-Cobelas et al. (2008, 2009) (Table 3).

**Table 3:** Area-specific export of total nitrogen (TN) and total phosphorus (TP) by various European, American, and world’s rivers (source: Howarth et al. 1996; EEA 2005; Radach and Pätsch 2007; Alvarez-Cobelas et al. 2008, 2009)

Region	Export to river basins		Export to coastal zones	
	MONERIS		Monitoring data	
	TN [kgN/km^2^/year]	TP [kgP/km^2^/year]	TN [kgN/km^2^/year]	TP [kgP/km^2^/year]
Vistula 1995–2015—this study	898	70	485	32
Oder 1995–2015—this study	921	54	512	30
Elbe (Radach and Pätsch 2007; EEA 2005)	1600	80	1015	50
Ems (Radach and Pätsch 2007; EEA 2005)	2900	240	1660	66
Rhine (Radach and Pätsch; EEA 2005)	2900	130	2181	163
Weser (Radach and Pätsch; EEA 2005)	1950	105	1469	80
Po (EEA 2005)	3600	140		
Danube (EEA 2005)	750	80		
Axios (EEA 2005)	500	300		
Daugava (EEA 2005)	700	40		
Baltic catchment (Howarth et al. 1996)			495	48
Northeast coast of the USA (Howarth et al. 1996)			1070	139
North Sea continental shelf (Howarth et al. 1996)			1450	117
Northwest coast of Europe (Howarth et al. 1996)			1300	82
Amazon basin (Howarth et al. 1996)			505	236
World’s rivers (Alvarez-Cobelas et al. 2008, 2009)			1–20,630	0.008–5100
In ca. 50% of observations of Alvarez-Cobelas et al. (2008, 2009)			0–2000	0–100

Highlighted by Alvarez-Cobelas et al. (2008, 2009), the need to use multiannual data to assess the impact of, e.g., anthropogenic pressure on the export of N and P, is clearly visible in nutrient management approach in the Baltic basin. In the light of historical and up-to-date findings defining ecological status of the Baltic Sea, Håkanson et al. (2010) and Pastuszak et al. (2018) suggest that proposed riverine TP load reduction in the Baltic basin is overestimated and its realization may lead to (i) undesirable consequences for the Baltic ecosystem and (ii) would require a decline in TP concentrations in Polish rivers to values reported for pre-industrial times. Concentrations of nutrients in the Vistula and Oder already meet the requirements of the Water Framework Directive (Garcia et al. 2012), and they are much lower than their counterparts in Western Europe (Bouraoui and Grizzetti 2011; OECD 2008) or in the Humber catchment in north-western England ^(^Neal et al. 2008). So, one aspect is the real ecological need of the Baltic ecosystem, and the other aspect is the establishment of an appropriate level of TP load reduction and national allocation of TP load reduction, which, in the opinion of Pastuszak et al. (2018), was based on too short reference period (1997–2003) (HELCOM 2013). Considerable difference between TP fluxes in relatively short and in a very long time span has been documented in these studies (Fig. 8), and in studies based on monitoring data (Pastuszak et al. 2018). This translates into not taking into account the reduction of TP fluxes in the period preceding the reference period, and, as a consequence, results in unworkable assumptions of TP flux reduction now.

In 1995-2015, average area specific N emission in the Oder basin was by 23 kg N km^-2^ year^-1^ higher, whereas area specific P emission by 16 kg P km^-2^ year^-1^ lower than in the Vistula basin (Table 3); higher N emission can be explained by more intensive agricultural activity (e.g. higher N surplus; Suppl. 10), whereas lower P emission can be explained by higher absorption of European Union funds and higher investment in WWTPs and agricultural infrastructure (Kata and Miś 2006; Kiryluk-Dryjska 2007; Kowalkowski et al. 2012.

### N and P retention in the Vistula and Oder catchment

In comparison with other large European rivers, the Vistula is rather unique as only short reaches have been regulated. It runs naturally over long stretches and water flow is quite irregular. The central stretch is particularly dynamic, with braided channels, permanent and temporary islands, and rich valley vegetation. The verdant embankments, wetlands, and swamps that are found along most of its course remove significant part nutrients that are discharged into the river; therefore, they are of considerable importance (Kajak 1992; Kristensen and Hansen 1994). The Oder River and most of its tributaries are also characterized by relatively low level of natural environment changes. The Oder catchment covers seven national parks, landscape parks, and nature reserves. Many areas in Poland, including the Vistula and the Oder valleys, are subject to special protection under NATURA 2000.

Stålnacke et al. (2015) showed that around 380,000 tons of N are annually retained in surface waters draining to the Baltic Sea, thus 100,000 tons more than estimated by HELCOM for the year 2000 (HELCOM 2004). The total annual riverine load from the 117 basins to the Baltic Sea was estimated at 570,000 tons of N, giving a total surface water N retention of around 40%. In terms of absolute retention values, three major river basins account for 50% of the total retention in the 117 basins, i.e., around 104,000 tons of N is retained annually in the Neva, 55,000 tons in the Vistula, and 32,000 tons in the Oder (Stålnacke et al. 2015). The mean retention values obtained by us are very close to those reported by Stålnacke et al. (2015), who used a different model and a different approach. Thanks largely to the natural course of the Vistula and Oder and their tributaries, over 91,000 tons of N and over 7600 tons of P are annually retained in the catchments of these rivers (Table 2). These estimates do not cover TN and TP retention in estuaries, e.g., Oder estuary with its largest component, i.e., the Szczecin Lagoon (Fig. 1), which retains 45% of the Oder TN load (ca. 37,000 tons N year^−1^), and 37% of the Oder TP load (ca. 2200 tons P year^−1^) (Pastuszak et al. 2005).

Enormous effort has been made to reduce N and P emission into the river basins and loads discharged from Polish territory to the Baltic Sea (Kowalkowski et al. 2012; Pastuszak et al. 2012a, b, 2018). Two things may cause an increase of N and P emission in the years to come, one generated by nature (climate change and more frequent events of flooding causing excessive nutrient emission, particularly nitrogen; see also Pastuszak et al. 2014), and the other one, a potential possibility of river channelization (Gawlik and Sufin-Jacquemart 2016). Elosegi and Sabater (2013) review the literature on the effects of common hydro-morphological impacts (channel modifications and flow modifications) on the function of the river ecosystems. They prove that even light hydro-morphological impacts can have significant effects on river ecosystem functioning, e.g., decrease in sediment, nutrient, and organic matter retention and decrease in ecosystem respiration and in secondary production.

## Conclusions

Growing anthropogenic pressure in the nineteenth/twentieth century, demonstrating in, e.g., enhanced nitrogen and phosphorus emission into river catchments and coastal waters, undoubtedly differently tackled in various countries on the globe. Mathematical models are essential to improve our understanding of the interactions between multiple processes in different landscape elements in river basins and to better anticipate the transfer of nutrients from the mainland to the sea. We discuss impact of long-term increasing and then decreasing anthropogenic pressure on both the overall N and P emission into Polish large riverine systems and the shares of nutrient emission pathways.

Model MONERIS has been used in numerous European river basins, the Vistula and Oder (Poland) included, and the historical outcome of the studies in the latter river basin is available for the years 1880–2000. Continuation of modeling studies till 2015 gave us a unique opportunity to comment on the response of riverine systems to (i) increasing anthropogenic pressure due to growing population, industrialization, intensification of agricultural activity, and recently to climate change and related extreme weather conditions (floods, droughts); and (ii) decreasing anthropogenic pressure resulting from nutrient mitigation measures implemented in agricultural sector and construction of a large number of WWTPs. All these changes are additionally documented in the Supplementary material.

The following conclusions can be drawn from our studies:

- Long-term scientific research is necessary to correctly assess the impact of time-varying anthropogenic pressure on N and P emissions into river basins and further to the sea. For example, without a long-term perspective, we could conclude that during the transition period, covering only the years 1995–2015, P emission into the Oder basin declined by ca. 2500 tons year^−1^, while the longer time span (1985–2015) proves that it decreased by more than 9000 tons year^−1^. Determining the appropriate level of emission reduction already obtained is of key importance in determining a feasible level of further nutrient reduction without deteriorating the goods and services of the terrestrial and marine systems.
- Population growth and agricultural intensification in the nineteenth/twentieth century were responsible for respective 5.3-fold and 3.5-fold increase in N and P emission into the Oder basin, with the maximum (135,000 tons N year^−1^; 14,000 tons P year^−1^) observed at the turn of the 1980s/1990s.
- We believe that available data allowed us to encounter the maximum of N and P emission into the Vistula basin (170,000 tons N year^−1^; 14,200 tons P year^−1^) which was found at the turn of the 1980s/1990s.
- International commitments, but also financial support from the European Union, gave impetus to pro-ecological changes in Poland during the transition period (since 1989) which covered various sectors of the economy including agriculture, environmental protection, and related construction of a large number of waste water treatment plants (WWTPs) (2580 WWTPs in 1999–2014, not to speak of a large number of domestic plants).
- As a result, in 1985–2015, N emission into Oder basin decreased from 135,000 to 94,000 tons year^−1^; P emission declined from 14,000 to 5000 tons year^−1^. In 1995–2015, N emission into the Vistula basin decreased from 170,000 to 140,000 tons year^−1^; P emission declined from 14,200 to 10,600 tons year^−1^.
- In both river basins, the declines of nutrient emission were mainly generated by groundwater, WWTPs, and overland flow; the relative shares of these nutrient emission pathways in overall nutrient emission were considerably changing over time.
- In 1995–2015, three pathways, i.e., groundwater, tile drainage, and to a lesser extent WWTPs, played a key role in N emission, and they were responsible for ca. 80% of overall N emission in both basins; four pathways, i.e., erosion, overland flow, WWTPs, and urban areas, played a key role in P emission and accounted for ca. 80% of overall P emission into both basins.
- Contribution of a given nutrient emission paths must always be referred to natural and anthropogenic factors, both having great impact on nutrient emission.
- Weather conditions have a huge impact on increased (floods) or reduced (droughts) N and P emissions. Particularly, nitrogen emission is susceptible to variable weather conditions and it is strongly enhanced by long-lasting wet periods. Drought conditions may variously influence nutrient emission; therefore, analysis of emission magnitude requires adoption of meteorological drought indices and monthly/seasonal mapping of the river basin.
- Polish, not channelized rivers proved to be a significant natural nutrient trap; over 91,000 tons of N and over 7600 tons of P are annually retained in the studied river catchments.

## Electronic supplementary material

ESM 1 (DOC 212 kb)
