# Supplementary material for: Long-term changes in nitrogen and phosphorus emission into the Vistula and Oder catchments (Poland)—modeling (MONERIS) studies
Source: Environ Sci Pollut Res Int. 2018 Aug 25;25(29):29734–51. doi: 10.1007/s11356-018-2945-7 (PMC6153654; doi:10.1007/s11356-018-2945-7)
Supplement: Supplementary file 1 — (DOC 212 kb) [file 11356_2018_2945_MOESM1_ESM.doc]

**Long-term changes of nitrogen and phosphorus emission into the Vistula and Oder catchments (Poland) – modeling (MONERIS) studies**

**Journal: Environmental Science and Pollution Research**

Marianna Pastuszak, Tomasz Kowalkowski^*)^, Jerzy Kopiński,

Andrzej Doroszewski, Beata Jurga, Bogusław Buszewski

^*)^ corresponding author: email: pinez@chem.umk.pl, phone: +48-56 665 60 64 fax: +48-56 611 4837

Suppl. 1 Precipitation and overall water outflow from Polish territory in 1975-2015 (left graph) and precipitation in the Vistula and Oder basin in 1995-2015 (right graph) (data source: Fal et al. 2000; GUS 1991-2017; BDL 2017)

Suppl. 2 Annual water outflows with the Vistula and Oder in 1988-2015 (data source: IMWM 1989-2001, 1990-2002, 2003-2016).

Suppl. 3 Source apportioned nitrogen emission into the Vistula and Oder basins in the sub-periods: 1995-2002; 2003-2008; 2009-2015 (*please note different scales)*

Suppl. 4 Nitrogen emission by the Vistula and Oder in 1995-2002, 2003-2008, 2009-2015 –percentage contribution of seven pathways (atmospheric deposition, overland flow, tile drainage, erosion, groundwater, WWTPs, urban areas)

Suppl. 5 Source apportioned phosphorus emission into the Vistula and Oder basins in the sub-periods: 1995-2002; 2003-2008; 2009-2015 *(please note different scales)*

Suppl. 6 Phosphorus emission by the Vistula and Oder in 1995-2002, 2003-2008, 2009-2015 – percentage contribution of seven pathways (atmospheric deposition, overland flow, tile drainage, erosion, groundwater, WWTPs, urban areas)

Suppl. 7 Population in urban and rural areas of Poland in 1946-2015 (source of data: GUS 1981-1989; 1991-2017)

Suppl. 8 Application of mineral fertilizers (nitrogen, phosphorus, potassium) (1988-2015) and manure (1990-2012) in Poland (source: graphs prepared and made available by Tamara Jadczyszyn from the Institute of Soil Science and Plant Cultivation in Puławy, Poland)

Suppl. 9 Number of farm animals (cattle and pigs) in Polish agriculture in 1946-2015 (source of data: GUS 1981-1989; 1991-2017)

Suppl. 10 Nitrogen surplus in the Oder and Vistula basin in 1960-2015 (left graph) and phosphorus input, output, and surplus in Poland in 1985-2015 (right graph); UAA – Utilized Agricultural Area [source: data for the years 1960-2000 - from Eriksson et al. 2007; other data – our calculations with application of OECD (2004, 2006) methodology and GUS (1981-1989; 1991-2017) data]

Suppl. 11 Volume of industrial and municipal water (requiring treatment and treated) discharged to the surface waters or ground over the years 1970-2015 (source of data: GUS 1981-1989; 1991-2017)

Suppl. 12 Nitrogen and phosphorus loads discharged from WWTPs to the surface waters or ground in the Vistula and Oder catchment in 1995-2015 (source: BDL 2017)

Suppl. 13 Water consumption in Poland for production, agriculture and forestry, and water supply network in 1950-2015 (source of data: GUS 1981-1989; 1991-2017)

Table S1. Correlation matrix between monitored total nitrogen (TN), total phosphorus (TP) loads and significant model’s inputs

|  | runoff [m^3^/s] | arable land [%] | tile drained area [%] | precipitation [mm/m^2^] | P from WWTP [kg/a] | N from WWTP [kg/a] |
| --- | --- | --- | --- | --- | --- | --- |
| TN load [t/a] | 0.75 | 0.66 | -0.77 | 0.39 | 0.72 | 0.75 |
| TP load [t/a] | 0.86 | 0.46 | -0.75 | 0.49 | 0.57 | 0.70 |
